# Practices and Attitudes That Enhance Music Engagement of Adult Cochlear Implant Users

**DOI:** 10.3389/fnins.2019.01368

**Published:** 2019-12-24

**Authors:** Kate Gfeller, Ruth MacMullen Mallalieu, Aleksander Mansouri, Gaelen McCormick, Renee Blue O’Connell, Jake Spinowitz, Bettina Gellinek Turner

**Affiliations:** ^1^School of Music, College of Liberal Arts and Sciences, University of Iowa, Iowa City, IA, United States; ^2^Department of Communication Sciences and Disorders, College of Liberal Arts and Sciences, University of Iowa, Iowa City, IA, United States; ^3^Iowa Cochlear Implant Clinical Research Center, Department of Otolaryngology–Head and Neck Surgery, Carver College of Medicine, University of Iowa, Iowa City, IA, United States; ^4^University Library, The University of Sheffield, Sheffield, United Kingdom; ^5^Independent Scholar, Brighton, MA, United States; ^6^Eastman School of Music, University of Rochester, Rochester, NY, United States; ^7^Independent Scholar, Charlottesville, VA, United States; ^8^Independent Scholar, San Francisco, CA, United States; ^9^Independent Scholar, Newburyport, MA, United States

**Keywords:** cochlear implants, musicians, patient-engaged research, problem solving, music training, self-efficacy

## Abstract

**Background:**

Cochlear implants (CIs) are auditory prostheses designed to support spoken communication in persons with severe to profound hearing loss. Many post-lingually deaf adult CI users achieve good speech recognition in quiet; unfortunately, CI technology conveys a degraded representation of pitch and timbre, essential components of music. Not surprisingly, most CI users achieve significantly poorer perception and enjoyment of music compared with normal hearing listeners. Anecdotal evidence indicates that this impacts music engagement, particularly singing and playing instruments requiring ongoing tuning to external pitches or producing intervallic ratios. Interestingly, a small cohort of adult CI users has shown remarkable success in recovering or developing musical skills, but their success is poorly understood. Greater understanding of their efforts and attitudes may suggest potential rehabilitative approaches for other CI users.

**Purpose:**

This article documented personal characteristics and experiences perceived to contribute to high level musicianship. Research questions included: (1) What forms of practice/experience have most contributed to (re)establishing satisfying music making? (2) What situations or musical tasks are most frustrating or challenging? (3) What attitudes, motivational factors, or forms of support help CI users persist in working toward improved music engagement?

**Methods:**

Qualitative and patient–engaged research methodologies were used. Our study involved a unique collaboration of six CI users engaged in high levels of musicianship and a researcher whose scholarship focuses on music and CIs. The CI recipients conveyed their experiences and attitudes regarding music and CIs through open-ended narratives. These narratives were analyzed using an integrative approach of inductive and deductive coding methods. The codes and themes that emerged through inductive methods were then organized within the Dynamic Problem Solving Model for Management of Music Listening Environments (Gfeller et al., 2019a).

**Outcomes:**

This paper provides reflections of six CI users who successfully engage in active music making, including on-going tuning to external pitches and ensemble participation. Their perspectives emphasize the importance of pre-CI music instruction, extensive practice and immersion in music listening and playing, persistence and self-efficacy, and problem solving skills that optimize music engagement, and suggest possible strategies useful to other CI users interested in improving music experiences.

## Introduction

Cochlear implants (CIs) are auditory prostheses designed primarily to support persons with severe to profound hearing loss in spoken communication. Many post-lingually deaf adult CI recipients achieve good speech recognition in quiet (Looi et al., 2012). Unfortunately, most CI users achieve significantly poorer music perception and enjoyment than they possessed before hearing loss (Drennan et al., 2015), though there is considerable variability among CI users for music perception and engagement. Music perception typically does not improve significantly as a result of mere CI experience over time (for reviews, see Looi et al., 2012; Limb and Roy, 2014), though adult CI users may improve perception and enjoyment of some aspects of music as a result of focused listening and training programs (Gfeller et al., 2001; Fu and Galvin, 2007; Looi et al., 2012). Unfortunately, training programs designed for adult CI users are not readily available outside of selective research protocols (Gfeller et al., 2019a).

Most research on music and CIs focuses on enhanced perception and appreciation as measured in controlled laboratory environments, not production (for reviews, see Looi et al., 2012; Limb and Roy, 2014). A few pediatric studies indicate that some pediatric CI users do enjoy music making (e.g., Rocca, 2012; Gfeller et al., 2019b), but singing or playing in tune to an external pitch is problematic (Xu et al., 2009; Gfeller et al., 2012). These data are interesting, but cannot be generalized to adults whose auditory experiences included many years of normal hearing. The phenomenon of adult CI users who play music has received very limited attention to date within the CI literature.

While active music making may be thought of as a pleasant avocation, music making also has implications for (re)habilitation. Studies with normal hearing individuals document experience-based plasticity associated with longer-term music making (e.g., Kraus and Chandrasekaran, 2010; Herholz and Zatorre, 2012; Moreno and Bidelman, 2014; Patel, 2014). Music making involves several sensory systems (e.g., auditory, visual, tactile), the motor system, and makes demands on a variety of cognitive processes. Multimodal interactions that occur in longer-term instrumental playing can lead to stronger plastic changes in auditory processing than training in the auditory modality alone (Herholz and Zatorre, 2012). Music making is also associated with the reward system through direct feedback, pleasurable sounds, and social rewards in group music making (Herholz and Zatorre, 2012: Patel, 2014).

Due to reduced activation of regions that process spectrally complex sounds, the impact of music playing on experience-based plasticity likely differs from that of normal hearing adults (Strelnikov et al., 2015). More studies are needed to understand compensatory strategies used by the brain to decipher distorted input concerning music (Strelnikov et al., 2015). The phenomenon of music making among CI users is a topic currently under-represented in the literature and poorly understood. Factors such as residual hearing and the integration of non-auditory systems in experience-based plasticity may play an important role in music making. With regard to reward systems, despite the degraded signal, some adult CI users do find some forms of music enjoyable, and actively choose to listen to music (Looi et al., 2012; Gfeller et al., 2019a).

In addition to limited research on music making, the point of view of adult CI users, themselves, is greatly under-represented (Plant, 2015). CI recipients have typically been tested for perceptual accuracy or queried about music enjoyment through closed-ended items driven by researcher interests. Researcher-driven studies contribute extensively to our understanding of CIs and music, and research and development toward enhanced device technology remains an important goal for the CI field. Expanding our inquiries to explore the priorities and experiences of CI recipients, themselves, could shed new light on strategies for optimizing music, despite the current technology imperfect for conveying music (Limb and Roy, 2014). One possible approach is patient-engaged research.

A growing trend, patient-engaged (a.k.a. patient-centered) research, acknowledges that patients possess extensive knowledge and important insights into their own conditions as a result of lived experiences (Clancy and Collins, 2010). By ignoring the patient perspective, we lose a valuable source of information. Patient-engaged research calls for the involvement of patients at every stage in research planning, facilitation, and dissemination, though the extent of patient input varies dramatically from one study to the next (Bardes, 2012).

Qualitative research approaches are also considered effective in emphasizing patients’ perspectives, exploring under-researched topics, and examining questions involving experiences in everyday life. This compliments research questions better suited toward controlled experiments (Bradley et al., 2007; Creswell, 2014; Mather et al., 2017).

A variety of approaches are acceptable in qualitative research (Creswell, 2014). In general qualitative research examines broad questions rather than *a priori* hypotheses through words rather than numbers. Analyses may include a combination of inductive and deductive methods. This involves line-by-line coding of meaningful units of text by one or more coders followed by more deductive processes in which the emerging codes are organized in relation to existing theories or models (Bradley et al., 2007). In qualitative research, large amounts of textual data are gathered, thus not all information can be included. Therefore, researchers aggregate data into a small number of themes (Savenye and Robinson, 1996; Creswell, 2014), which are reported in narratives, often within an organizing model, and with liberal reporting of the participants’ own words (Bradley et al., 2007; Creswell, 2014).

The current study used qualitative and patient-engaged research methodologies to examine the phenomenon of adult CI recipients and music making. Rather than reporting on musical experiences of a broad cohort of “typical” adult CI users, it conveys the experiences of a select subsample of CI users who have achieved remarkable levels of musicianship. At present, high levels of music making by CI users is both rare and poorly understood. To better understand this phenomenon, the following research questions were examined:

(1) What forms of practice/experience have most contributed to (re)establishing satisfying music making?
(2) What sorts of situations or types of musical tasks are the most frustrating or challenging?
(3) What attitudes, motivational factors, or forms of support help CI users persist in working toward improved music engagement?

The life experiences of this group may offer insights regarding factors that contribute to extraordinary CI benefit.

## Materials and Methods

### Approach

The overall approaches were qualitative and patient-engaged research methodology. The CI users were involved in the study conceptualization, selection of research questions, methodological choices, contribution of data, review of the analyses, and preparation of the manuscript.

### Participant-Co-authors

The conceptualization of this paper evolved as a result of discussions at a symposium, The 2nd Music & Cochlear Implants Symposium, August 20–21, 2018, Montreal. The six musicians with CIs who collaborated on this paper discussed their musical experiences as part of a panel. These musicians were initially identified by the symposium organizing committee as possessing extraordinary musical skills. These skills were demonstrated at the symposium through videos or live performances, including improvization in a jazz ensemble. Their skill set includes singing or playing in tune on instruments (see Table 1) that do not have fixed pitches, which requires on-going tuning in solos and ensembles.

**TABLE 1:** Hearing profiles.

	**AM**	**ROC**	**JS**	**BT**	**RM**	**GM**
I play	Trombone	Guitar Flute/various wind Percussion	Guitar	Voice Piano Baroque recorder Fiddle/Ukulele	Clarinet	Double bass
Age	23	60	28	64	32	48
Gender	M	F	M	F	F	F
Status	Professional	Semi-professional	Amateur	Professional	Amateur	Professional
My musical training prior to implantation	Trombone lessons from middle school	High school percussion band and music theory. Private guitar lessons throughout twenties	None	Recorder, violin, piano lessons from age 7. University degree in music education. More than a decade of voice lessons; 45 years’ experience of choral singing	Recorder lessons from age 6. Clarinet lessons from age 8, reaching ABRSM^∗^ Grade 6 and Grade 5 theory. Played in orchestra and wind bands	Classical music student from age 9. Undergraduate degree in music performance from Eastman School of Music. Masters degree in music performance from Carnegie Mellon University
My musical training post-implantation	Weekly trombone lessons from high school, played in band. Undergraduate degree in trombone performance, University of Delaware. Currently studying for a Master of Music at Boston University.	Private lessons for Galax dulcimer (wind instrument from Appalachian region). Folklife apprentice for the Virginia Foundation of Humanities. Private lessons for aural rehab purposes after CI activation, with focus on pitch and interval recognition, sight singing.	Started playing guitar after implantation. Regular composition and jamming sessions.	Weekly piano lessons and occasional voice coaching. Teach students in piano and recorder. Continue to sing in small choir.	Started regular lessons aged 30. Currently trying to finish ABRSM exams. Voice coaching and singing lessons for past year.	Continue to use knowledge and methods from prior training. Teach double bass students and collegiate music-related courses.
Hearing loss	Post-lingual, bilateral, severe. Caused by enlarged vestibular aqueduct. Left ear: sudden loss, around 5 years of age. Right ear: more gradual loss over some months, aged 12	Post-lingual, bilateral, progressive, likely from birth but detected in twenties. Glandular fever, aged 21, caused further deterioration	Prelingual, bilateral, severe/profound loss from birth. Residual hearing deteriorated sharply aged 15	Post-lingual, bilateral. Gradual, progressive loss from forties, from high to mid frequencies	Prelingual, bilateral, sensorineural profound loss from birth	Post-lingual, bilateral. Caused by Ménière’s disease. Left ear: sudden onset deafness, aged 35, leading to profound loss by age 40. Right ear: Sudden onset Ménière’s at age 43, leading to moderate to profound hearing loss by age 45
Age implanted	L: 13 R: 18	L: 50	R: 15	L: 63	R: 13 L: 23	L: 47
Cochlear implant make and model	Cochlear Nucleus 6 with long electrodes	Cochlear Nucleus 6 (electrode information not known)	Cochlear Nucleus 7 (electrode information not known)	Advanced Bionics CQ 90 EAS with HiFocus Slim – J electrode, full length	MED-EL Sonnet with COMBI 40 + with STANDARD electrode (R) and SONATA with FLEX SOFT electrode (L)	Advanced Bionics Naida Q90 (electrode information not known)
Additional devices used	N/A	R: ReSound hearing aid	L: ReSound hearing aid	R: Phonak Naida Link hearing aid	N/A	R: Widex, Beyond 440 hearing aid

**^∗^ABRSM, Associated Board of the Royal Schools of Music exams.**

Consistent with qualitative methodology and case studies, this was a purposive sampling of individuals who possess characteristics relevant to the questions at hand. These individuals present a small “community” of shared life experiences: CI users who share a deep passion for music, whose daily lives involve many hours of engagement in music listening and playing, characteristics rare among CI users (Looi et al., 2012; Drennan et al., 2015).

Table 1 indicates a mix of professional musicians and musical avocation that requires considerable proficiency with tuning and production of pitch patterns well beyond the typical range of perceptual capabilities reported in the CI literature (for reviews, see Looi et al., 2012; Limb and Roy, 2014). Hearing history and musical background of the CI users appears in Table 1. As a group, they represent 182 years of musical training and production, and 52 years and 10 months of cochlear implant use.

### Development of the Research Questions and Interview Items

Qualitative research utilizes broad questions which are oriented toward participant perspectives (Creswell, 2014). The three overarching research questions addressed in this study were selected by the 6 CI co-authors as priorities for exploration. The first author presented the group five possible broad questions based upon her 30 years of experience in the field, and also requested that the CI users propose additional questions for consideration. The CI users independently ranked the pool of questions by priority and the group’s three top ranking questions were chosen; no additional research questions were suggested.

Consistent with qualitative methodology, the experiences of these CI users were gathered through open-ended inquiries, which tend to yield more detailed and personal perspectives than is typically yielded by quantitative studies using closed-ended items (Creswell, 2014). The co-authors, who live in 7 different locals, including 2 countries discussed via e-mail a protocol for gathering individual narratives followed by an interactive on-line focus group. The collaborators chose the first author to coordinate the research process, collect their narratives, to analyze the data, and to serve as “narrator” or primary writer of the study.

### Data Collection

Following a review, the IRB committee of The University of Iowa waived the need for ethics committee approval of this study. However, in the spirit of fully informed consent, each CI recipient was also sent a formal invitation via e-mail to participate in the online questionnaire; the decision to complete the questionnaire constituted formal consent. Because the identity of the CI recipients is revealed in their capacity as co-authors, each co-author also signed and sent the first author a document indicating the desire to be listed as a co-author.

After formal consent was obtained from each CI user, the first author e-mailed each co-author the three research questions and instructions for completing the questions as a word document. On-line inquires rather than in-person focus groups were used because the group members lived in distant locations and because written responses also reduced the possibility of errors in transcribing oral accounts (Tates et al., 2009; Creswell, 2014). Responses were completed independently and each word document was returned to the first author for consolidation into a master document with the responses from all 6 participants; individual responses were identified by alphabetical letters. In a second round of data collection, each CI user independently reviewed the consolidated document and commented on all responses with corrections or additional thoughts. This process commonly used in qualitative research, called member checking, allowed for verification and validation of data accuracy, and facilitated a more interactive component to data collection. The first author entered responses for the first and second rounds into a master word document for subsequent coding. All responses were downloaded into a password-protected database for analysis.

## Data Analysis

The analysis involved an iterative and integrative approach, utilizing a combination of inductive and deductive coding (Bradley et al., 2007). The interview data were first read carefully multiple times by the first author to get a general sense of the data. Each participant’s responses were analyzed independent of the three research questions because responses can apply to multiple questions. A line-by-line analysis was completed in which units of meaning (words, phrases, and sentences) were tagged or represented with an identifying code. The initial codes emerged inductively into like categories, with a total of 329 codes assigned to the narratives from the first two rounds of coding. Some sentences or paragraphs were assigned more than one code. Therefore, the percentage of codes that fit into categories exceeded 100%.

After the initial inductive coding, the codes were then grouped into more abstract, high level categories referred to as themes (Tates et al., 2009). Magnitude coding (frequency of codes), which can help determine the most prominent themes (Saldaña, 2013), was also used (see Tables 2–4).

**TABLE 2:** The frequencies of codes assigned to each theme/component of the DPSM model in rank order.

**Theme within the DPSM**	**Frequency of items (+ or – if applicable^∗^)**	**% of 329 total codes**
Problem solving skills total	115	35%
Problem solving orientation	107 (86+, 21-)	32.5%
Music, Music and Speech	67	20%
Social context	54 (32+, 22-)	16.4%
Auditory profile	52	15.5%
Transfer of past knowledge	49 (42+, 7-)	15%
Change over time	34	10.3%
Domain specific knowledge	22	6.6%
Environment	11	3.0%

**^∗^+ refers to positive or helpful factors; – refers to factors that impair music listening.**

**TABLE 3:** Code frequency and examples for problem solving skills component.

**Theme: skill or strategy**	**Frequency**	**Examples**
Use of all senses	23	Visual, tactile, muscle memory, proprioception, movement
Top down processing	14	Using memory of musical sounds, imagination, internal sense of pitch
Selecting most accessible sounds	12	Latching on to most accessible sounds (e.g., best pitch range, best quality sound) as strategy for satisfaction or as jumping off point to extend skills
Focus and energy	11	It takes focus and energy to listen to music and improve; hard to do when tired
Music theory types of exercises	11	Many hours doing ear training exercises similar to what one learns in theory, such as interval training, using a fixed pitch, listening to sequences, listening for subtle pitches, applying pitches onto prior knowledge of songs
Extensive and focused listening to music	10	Taking many opportunities to listen to CDs, music on line, repeated listening for various layers of music
Extensive making of music	8	Practice a lot, playing in rehearsals, making music offers foundation for learning about music
Post-implant music lessons	6	Benefitted from guidance, motivation, specific exercises from theory or studio teachers
Social learning	5	Importance of input from other CI users at conferences, support groups, on line. More important than input from hearing professionals
Speech training exercises, listening to speech	5	Carry over of speech training to music, listening to different dialects, accents
Singing	4	Singing helps with intonation
Technology	4	Use of headphones, tuning apps, synergy of CI with HA

**TABLE 4:** Code frequency and examples for problem solving orientation component.

**Problem solving orientation**	**Frequency of codes**	**Examples of codes**
Problems as an opportunity, a challenge	44	Enjoying a challenge, seeing problems as opportunities to learn more, seeing every music experience as a chance to learn more, hard work can be fun, life long learning, energy is important
Cognitive reframing	14	Being able to find the positive in a situation, such as realizing everyone hears differently, being realistic no one can do everything, focusing on what I can do, CI experiences resulting in an interesting life
Music as a passion and motivation to strive	9	Love of music, sense of identity, keeps one working, love of sound
Social component	7	There is a social component to striving; connecting with others at conferences, participating in research, networking with others, helping others
Positive expectancies	4	Belief things will come together over time, improvement with speech gave hope for improvement with music

**FIGURE 1:**
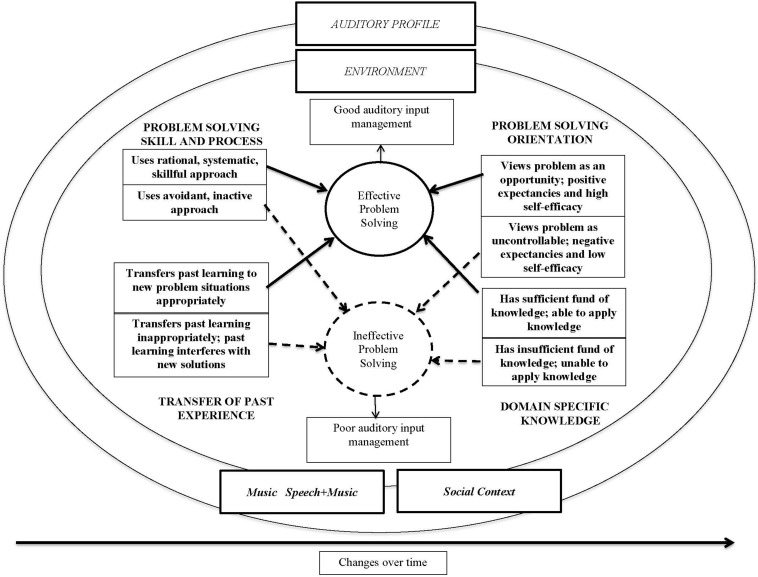
Dynamic problem solving model for management of music listening environments.

Consistent with an integrative approach, the interaction of coding and themes that evolved from inductive coding were then examined deductively in relation to existing models and theories. The model chosen as the best fit was the Dynamic Problem Solving Model for the Management of Music Listening Environments (DPSM) which is described in detail in Gfeller et al. (2019a) (see Figure 1). This model was initially developed to conceptualize the music experiences of a broad range of adult CI users.

The DPSM guided the deductive stage of analysis for this study, which is described in the Results. The components of the DPSM is described more extensively in Gfeller et al. (2019a) (see Figure 1). Briefly, the outer elliptical components of the model (auditory profile, environment, music, music and speech, and social context) reflect the reciprocal processes associated with music listening responses. The listener’s management of the music experiences appear within the ellipses: cognitive, affective and behavioral processes associated with self-efficacy, problem solving and self-management. Changes over Time emphasizes the dynamic and reciprocal nature of the music, listening environments, and listener attributes and actions (e.g., experience-based plasticity, changing attitudes and behaviors).

As part of verification and validation of the analyses associated with qualitative methods, 20% of the codes were reviewed by a second coder, a laboratory associate uninvolved in this study. Agreement between coders was 75%. Differences in coding choices were resolved through discussion. The results were then shared with the 6 CI users through member checking to validate that the themes reflected their responses (Creswell, 2014).

## Results

Table 2 presents in rank order the frequencies of codes assigned to each theme/component of the DPSM. Greater frequency, and thus larger proportion of the entire data set, suggests that an idea is more prevalent and important to the target group (Saldaña, 2013). Each of the themes represented in the DPSM is described in greater detail below in order of greatest to lowest frequency. While these themes and exemplars are presented as discrete categories, in real life, these factors often interact and overlap.

### Problem Solving Skills

This theme includes specific strategies used for improving music experiences. A total of 115 out of 329 codes were assigned to this category. The precise and technical descriptions of strategies revealed a high level of technical knowledge regarding music theory and music pedagogy. Strategies differed somewhat as a function of the instrument played (e.g., relying on vibrotactile feedback from bass, guitar, or singing), prior background (e.g., formal theory instruction), and onset of hearing loss (e.g., internal representation of musical sounds developed through natural hearing).

Table 3 presents the skills or strategies in rank order of frequency. The strategies or methods used as part of training included accommodations (working around limitations), compensatory strategies (e.g., synergistic use of several sensorimotor inputs to compensate for degraded auditory input), and focused practice on music components (e.g., a semitone) that resulted in perceptual changes over time (e.g., being able to hear a semitone change, matching an external pitch).

Several individuals emphasized the benefit of multisensory input: “I believe that engaging all of the senses is very helpful to regain music perception.” “Reading visual notation supports perception of what I am hearing around me and informs me what sounds to expect.” More accessible sounds were also chosen for practice or greater listening satisfaction: “In choosing pieces for myself, I avoid music that contains a lot of thick, complex chords.” “Practicing with a fixed sound medium.” Given current limitations of accessible music training materials that are designed for use by adult CI recipients (Gfeller et al., 2019a), the extensive codes in this category suggests a high level of personal ingenuity in finding resources.

While Table 3 offers a rich menu of possible strategies for enhancing music, it is notable that many are based upon formal music theory or approaches that require reading notation or some level of music understanding.

“I worked with a music teacher and did ear training/pitch recognition and sight singing exercises. My teacher would play two pitches either in succession or simultaneously and I would have to try and discern what interval she played. Things like major 3rd, perfect 4th, perfect 5th, octave, etc… Note that all of these exercises are done in basic music theory classes”.

Some strategies required a commitment of time that could be considered excessive by some CI users: “It is not because we spent an occasional hour on a website aiming to improve our music perception post-CI. It is the immersion in musical pursuits that produces extraordinary results.” This brings to mind another prevalent characteristic of these CI users: a strong problem solving orientation, which is the focus of the following section.

### Problem Solving Orientation

A problem solving orientation includes personal characteristics such as viewing problems as opportunities, cognitive reframing (finding a positive interpretation to negative events), hardiness and persistence, enjoying challenges, and high sense of self-efficacy (confidence in accomplishing one’s goals) (Hill-Briggs, 2003). Limited problem solving orientation includes seeing problems as uncontrollable, having negative expectations of outcomes, and low self-efficacy.

Problem solving orientation was the second highest (107) model component in coding frequency (see Table 4). The vast majority (86) of the codes revealed a positive problem solving orientation. Several described themselves as a “lifelong learner” and emphasized persistence, hardiness and embracing challenges: “I challenge myself by listening to new music.” “I believe that the key to improvement is within myself.” “Be tenacious, persistent, curious, patient, understanding, open-minded, gentle and kind to yourself. Also have fun! Learning is fun and having a CI is a unique experience few get to have.”

Examples of being able to focus on positive aspects (cognitive reframing) included: “I am trying to remain ‘fascinated by the process’… and not get overwhelmed by the vast number of things I cannot hear anymore.” “We must look for the good days rather than linger on the bad ones.” “understanding that the rate at which you improve does not reflect whether you are a good person or ‘good at music’.”

Considering motivational factors that fueled a positive attitude, a strong passion for music was central to this attitude for several in this cohort: “Music has always been my greatest source of joy and I was determined to never give up on it or lose it.” “The most important attitude component for helping with CI music perception is a personal passion for music and strong disposition to stick with music engagement even when the quality might not be satisfactory at the start.” “I love sounds. Anything that can be heard is interesting to me, and hearing it better becomes my goal.”

While all CI users showed a strong problem solving orientation, statements of frustration and lower self-efficacy tended to be expressed by the individuals with less than one year experience at the time of data collection. However, those CI users with greater length of device use also reflected back on difficulties they experienced in the early months following implantation. The first 6 months post-implant seemed particularly problematic. “For about 6 months, music sounded absolutely horrible and I was afraid I might never enjoy it again. But I persisted.”

Among codes that reflected lack of control were frustration with chaotic and unpleasant sounds, feeling lack of control over sense of pitch, and needing to avoid some musical situations (singing with others, playing in large ensembles) because of difficulty and lack of emotional reward. One musician with less than one year’s CI experience and some residual hearing noted “Listening to music is almost uniformly uncomfortable for me now, [with] the pitch distortion which makes all music sound chaotic.” Avoidance as a strategy tended to occur more frequently in early months post-CI.

### Music and Music and Speech

The third most frequent component (20%) addressed structural features of music or music in conjunction with speech. Perception of the structural features of music seemed to be particularly problematic during early CI use; 61% of the codes were contributed by CI users with less than one year’s CI experience.

Within this category, difficulty with pitch made up 25% of all codes. This is not surprising given the characteristics of CI technology in relation to music (Limb and Roy, 2014). Problems regarding pitch included: establishing an internal sense of pitch, difficulty hearing pitches in higher and lower ranges, poor error detection, problems hearing key changes in the harmony, matching an external pitch, confusion of major and minor, or hearing a discrepancy between acoustic and electric stimulation (e.g., the pitches being a half step “off”). Problems with pitch seem to be particularly difficult in the early months of CI use, and resulted in a chaotic and distorted sound. As will be described later under “Changes over Time,” several CI musicians experienced improved pitch perception, but this required many months of listening exercises.

Given the difficulties with pitch perception, it is unsurprising that multi-layered music (music with harmony or counterpoint) was a common subtheme (19%) of this category. Participants described separating out the parts of large vocal or instrumental ensembles as difficult or nearly impossible. This likely contributes to another sub-category of problem associated with music: playing or singing in ensembles with other musicians (9%). A participant with less than one year’s experience stated, “It is very difficult for me to hear all voices and harmonies in a full ensemble, plus accompaniment if applicable. My experience can easily turn into what I call ‘soundsoup.”’

Fifteen percent of the codes in this category described some instruments as more or less accessible and pleasant for CI recipients. One musician found using the piano in exercises to train pitch patterns and harmonies beneficial. Another described the resonance and wide frequency range of the clarinet as particularly helpful in accessing musical sounds. Most of this cohort plays instruments without fixed pitches: singing, playing guitar, bass, and trombone, which require ongoing tuning.

Other problem with music or speech included overall poor sound quality, unpleasant distribution of overtones, and an annoying overlay of noise in the CI signal. One musician described difficulty hearing the conductor’s voice over the ensemble, and several described difficulty hearing music or speech against background noise, which is related to the social aspects of music making.

### Social Context

Music making or listening to music often involves collaboration and shared learning. Music concerts and music-making often bring people together for aesthetic enrichment or entertainment (Hargreaves and North, 2010). In this dataset, social context comprised 16.4% of the total number of coded items. The most prominent sub-category (24% of items in this category) was encouragement and input from other CI users, especially those with musical background and interests. This cohort connected with other CI users on line, in support groups, and at conferences; these connections formed their most important source of motivation as well as information. Support of friends, family, and teachers were also noted: “having a supportive network of musicians is very helpful.” “The people in my life. always encouraged me to pursue music.” Good teachers were described as an important source of understanding, guidance, and motivation.

Even though the incidence of hearing loss is fairly common among musicians, the most common negative commentary on social context was stigmatization of CI users by other musicians (14.8%). “Being dismissed as a musician due to deafness or CI happens occasionally and is difficult to accept.” “We stigmatize those with hearing loss in the pro music community, as if they were somehow at fault.” Because of these social concerns, 3 of the 6 CI users now make efforts to inform others about CIs and music and to dispel myths.

### Auditory Profile

This theme addresses hearing history (e.g., age of onset, residual hearing, CI or HA use, etc.) as well as music experiences that contributed to auditory development; it comprised 15.5% of all codes in the dataset.

The limitation of the CI for conveying musical structures was the primary sub-category (24 codes), including problem with sound quality and limitations for pitch and overtones: “Post-CI, my main issue has been a constant ‘overlay of extra sounds’ ….” “The problems associated with the hearing loss, recovery, and day-to-day use of hearing aids, implants, or both are draining.” Several commented that they need considerable energy to enjoy music through a CI.

Four of the participant co-authors use hearing aids to optimize residual hearing. A primary sub-category of this theme was the synergistic benefit of residual hearing (including bimodal hearing). “The bimodal set up (HA + CI) helped as I could still follow along with music reasonably well through the hearing aid even when the CI perception wasn’t clear.” As persons with technical knowledge of music, several were able to describe in precise terms inconsistencies between acoustic (residual hearing) and electric hearing, as well as resolution of those inputs over time. Music training before implantation (as part of hearing experiences) was described as an important foundation for learning music with the CI. One person utilized their memory of pitches from when they still had “natural hearing” before receiving the second implant as part of “relearning” correct pitches and intervals after receiving a second implant.

### Transfer of Past Knowledge

This theme within the model makes up 15% of all the coded items. Five of the cohort had many years of formal music instruction before implantation, and 3 are professional musicians. Transfer of knowledge from prior vocal or instrumental music instruction (30.6%) included use of non-auditory cues (visual, tactile, proprioceptive), understanding the building blocks of music, work ethic, and discipline from taking lessons prior to hearing loss, being trained to listen for subtleties, and knowing strategies for collaborative music making. “In my formal music education, we sometimes talked about ‘feeling the string’ through the bow’ contact with the hair on the string. I had taken that farther to feeling the string through my fingers contact of the bow.”

This cohort transferred knowledge of theory or ear training strategies extensively toward optimizing CI use (28.5% of codes in this category). This included knowledge of pitch relationships, theory exercises, reading notation, and internal sense of pitch or timbre learned through natural hearing. Theoretical understanding of music provided a rich mental representation of music that fosters top-down processing, which contributed to the re-establishment of more normalize pitch percepts.

Several described a transfer of speech training to music. For example, “It was regular auditory training for baseline speech perception that formed the bedrock for my ability to appreciate music with the CI… once the vocals (lyrics) were formed as the baseline for what I could latch on to, other elements started to follow with time and persistence.”

In some instances (14.3%), past knowledge did not carry over effectively to music making after implantation. For example, in collaborative music-making, the CI user could no longer rely on the overtones for creating sound quality. Given the degraded percepts of pitch and timbre, some described a period of exploration to find new strategies.

### Changes Over Time

Changes over time were referred to directly in 10.3% of the coded responses. This included description of the first 6 months of CI use being especially bad, gradual improvement with time, or the many hours spent in practicing or listening. However, one can argue that passage of time is implied in most every aspect of the phenomenon of restoring musicality, and thus is an important addition to this model. Experience based plasticity, such as integration of multimodal input associated with ear training or music performance requires sufficient exposure to sound and repetition of tasks to support learning/plasticity; repetition occurs over time and neural changes take time (Herholz and Zatorre, 2012).

As one of the musicians described, “I chose to sit at a piano and play things [patterns of notes] until they sounded different. At first, the keys C and G, a perfect fifth interval, sounded the same.” Over time, this musician was able to hear musical scale patterns, match pitches through singing, and eventually created correct pitches on trombone, which requires ongoing tuning.

Furthermore, music is a time-dependent art form; it involves combinations of pitch, timbre, rhythm, and amplitude that change rapidly over time. Changes such as hearing loss or implantation also may include a course of acceptance and adjustment that can affect problem-solving orientation (Gfeller et al., 2019a). As a more recently implanted participant noted, “I have not reached a level of ‘satisfying music making’. but have reached a level of ‘transitional acceptance,’ which means I am still hoping for improvement.”

Specific to this cohort of CI users, the most prevalent sub-theme was the amount of practice time required to achieve satisfactory outcomes (35.3%). Half of the CI users described themselves as life long learners, thus suggesting their musical quest does not have an end goal/destination. Another common theme was that music sounded really bad and chaotic at first (23.5% of codes), but as many (23.5%) codes indicated music gradually improved with time and effort.

### Domain Specific Knowledge

This component of the model refers to knowledge of hearing loss, CIs, music, or combinations of those elements, and comprised only 6.6% of all coded comments. Despite the fact that 5 of the 6 CI users had many years of formal music training, there was limited reference to having specific knowledge in these areas. Musical knowledge was revealed indirectly through particular forms of knowledge, such as comments about distorted overtones in the CI signal. Twenty of the 22 codes referred to knowledge of music theory or understanding of the impact of hearing loss or CI use on music perception.

### Environment

This theme represented within the model refers to issues such as poor room acoustics or competing noise in the environment – that is, the surrounding conditions in which music is heard. It made up a very small proportion (3.3%) of all codes for the CI users. Four of the CI users described problems of rehearsing in small rooms. “Playing in small venues is bad because the sound becomes one big mess during loud sections and directions are near impossible to hear.” The acoustical environment and background noise also undermined enjoyment when attending concerts.

## Discussion

In considering the various themes represented in the DPSM, the strong problem solving orientation and ingenious approaches to problem solving are among the most notable characteristics of this group of CI users. Particularly impressive is the immersion and intensity with which these individuals have tackled the perceptual problems associated with accessing music through a CI. The capacity to persist with hours of listening and playing exercises despite annoying sound quality, no guarantees of eventual benefits, and very gradual improvement requires patience and fortitude.

While avoidance has been described in models of chronic illness as a less effective problem solving strategy (Hill-Briggs, 2003), avoidance may sometimes have adaptive value after initial hookup. More positive experiences were sometimes characterized as gradually “earned.” Some started with the most accessible sound as a foundation for interpreting the signal, then eventually seeking more challenging situations. This might be likened to the behavioral technique of successive approximations, beginning with a simpler task that can be achieved and gradually building upon small successes. Thus, temporary avoidance may in fact be a realistic problem solving strategy in the first months after implantation. The problem solving orientation is likely a key to persisting from initial frustrations toward gradual improvement.

The experiences of this cohort is an interesting contrast to comments from a more diverse and “typical” group of 40 CI recipients described by Gfeller et al. (2019a), who represented a wide range of musical background (no musical training to college level instruction), interests and perceptual abilities. The “typical” group’s average length of CI use was 12.25 years (2.44–28.07 years). In response to a question regarding potential interest in music training, the more “typical” group expressed a strong interest (90%) in music training as long as the training did not require more than 30 min a couple of times for a total length of 1 or 2 weeks. Because experience-based plasticity requires extensive repetitions or exposures, and CI technology is not inherently suitable for conveying fine structure, it is unrealistic that a CI user would be able to accomplish high-level musical skills as a result of a few hours of effort. However, prestige musicianship is also not a strong priority for all CI users.

This group of CI musicians offers an amazing profile of what can be accomplished despite a degraded signal. This group also shines a light on the impact of attitude and motivation as central to rehabilitation. Music is a passion and part of their identities. Thus, they are willing to invest immense effort and time to restore some musical enjoyment. Without this level of passion, such extensive effort is likely to be viewed as too time consuming or discouraging. This remarkable group does not represent the more typical profile of CI users on several factors, including age range, extent of residual hearing, extensive and formal musical training before or after implantation, and level of motivation; consequently, hearing professionals should make a strong effort to clearly understand the motivations, background, and aspirations of CI users before establishing unrealistic expectations for music enjoyment and rehabilitation. Nevertheless, these individuals demonstrate the impact of on-going focused training, and they offer possible strategies that might be used toward more modest levels of improvement.

In addition to immersion and motivation, the CI musicians also tended to focus on their internal capabilities for improving the sound and altering their auditory percepts to a greater extent than more typical CI users (Gfeller et al., 2019a). The CI musician group focused very little on technological options. As one user put it, “I think we can get too hung up on what we can hear – for example by mapping implants.”

Only 3.3% of codes from this cohort addressed the problems of a noisy or acoustically difficult environment. The more diverse CI group reported by Gfeller et al. (2019a) tended to emphasize barriers to music enjoyment from the environment, situation, or CI technology. Their problem solving strategies tended to take the form of accommodations or avoidance of problematic sounds as opposed to rehabilitation or restoration. The more “typical” CI user, however, also does not possess the wellspring of musical training and resources of these musicians with CIs; consequently, accommodations or compensatory strategies may be a more realistic approach for less motivated CI users in managing complex listening situations.

How might the experiences of this unique group inform other CI users or their hearing professionals? A review of the problem solving skills listed in Table 3 and problem solving orientation in Table 4 offer some possible strategies, exercises, accommodations, and behavioral or attitudinal approaches that could act as a menu of options to consider if a CI user is interested in more satisfactory music involvement. As the DPSM indicates, a number of factors – auditory profile, type of music, environment, personal characteristics – can interact and change with time and circumstance. Thus, various options will be more suitable for some individuals than others, as well as for particular listening circumstances. Satisfactory engagement in music is likely to require some basic knowledge paired with flexible and dynamic problem solving.

In summary, the experiences of this cohort of CI musicians represents unusually high levels of domain-specific knowledge paired with a strong problem solving orientation and flexibility in applying problem solving skills. They have utilized accommodations, compensatory strategies, and training to enhance perception with end results that exceed expectations, based upon technical features of the device. These CI users might be referred to as auditory athletes, who like high-level athletes, push their capabilities to extreme levels. Comparisons come to mind with stroke patients who have pushed through months of painstaking rehabilitation, with no guarantees of outcomes, to restore motor or cognitive functions (Brown et al., 2014). While more typical CI recipients may have a less robust problem solving orientation and fewer resources upon which to build, this model nevertheless provides a framework for considering those factors needed for a given CI user to optimize their daily experiences with music listening or music making. These data also suggest some interesting avenues for future hypothesis testing to explore experience-based plasticity, compensatory strategies, motivation, and other internal factors that impact CI benefit.

## Data Availability Statement

The raw data supporting the conclusions of this article will be made available by the authors, without undue reservation, to any qualified researcher.

## Ethics Statement

Ethical review and approval was not required for the study on human participants in accordance with the local legislation and institutional requirements. The patients/participants provided their written informed consent to participate in this study. Written informed consent was obtained from the individual(s) for the publication of any potentially identifiable images or data included in this article. For further information, please see the author’s correspondence with the IRB in the Supplementary Material.

## Author Contributions

KG coordinated data gathering, management, coding, and analyses. RM, AM, RO, JS, GM, and BGT contributed narratives regarding the music experiences of CI users. KG and RM addressed guidelines for IRB approval. RM gathered and analyzed the data for Table 1. KG served as primary writer for the manuscript, though all authors contributed to article content, and reviewed analyses of the data and article content. All authors contributed to the conceptualization of the manuscript and protocol for gathering information.

## Conflict of Interest

The authors declare that the research was conducted in the absence of any commercial or financial relationships that could be construed as a potential conflict of interest.
