# Supplementary material for: Practices and Attitudes That Enhance Music Engagement of Adult Cochlear Implant Users
Source: Front Neurosci. 2019 Dec 24;13:1368. doi: 10.3389/fnins.2019.01368 (PMC6937904; doi:10.3389/fnins.2019.01368)
Supplement: Supplementary file 1 [file Data_Sheet_1.PDF]

To: the Editors of the Special Edition on Music and Cochlear Implants for Frontiers in Neuroscience

From: Kate Gfeller, Ph.D. The University of Iowa

Re: Submitted Manuscript, Practices and Attitudes that Enhance Music Engagement of Adult Cochlear Implant Users

As you are aware, I am working with the six cochlear implant user-musicians who were panelists at the Montreal Symposium on CIs and Music in August, 2018. Discussion during the symposium lead to a 'group' decision that I would work with the CI users-musicians to write a paper that describes their experiences and offers insights into strategies, attitudes, and other factors that have resulted in their remarkable success in making music. We have worked collaboratively over the months to produce what we hope is a valuable contribution for *Frontiers.* Our preliminary abstract was accepted for further consideration, and we are ready to submit our manuscript. I am writing this letter to alert you to some logistcial issues and our efforts to meet scientific guidelines for publication of research involving humans.

The issue is compliance with regulations for research with human subjects. One of the common requirements for any research involving human subjects is the ethical promise of confidentiality—that participants cannot be identified or linked to specific data points. We fully understand that these guidelines are based upon important ethical values. However, our submission represents patient-engaged research methodology, and I am eager that the CI recipients be able to speak in their own voices (as they wish to do as well) rather than me writing as an observer of their behaviors.

The interpretation and implementation of the rules in the case of patient-engaged research can be complex. A patient may contribute to such an extent to merit acknowledgement as an author. That is the case in this study. The CI users from the panel have been engaged in the research decisions throughout our process. While I have taken leadership in facilitating data collection, analyses, and write up, the CI cohort has been integrally involved in the conceptualization of the process and the sharing of narratives.

This manuscript is unique in that the data regarding the music experiences of adult CI recipients have been shared by six of the co-authors of the manuscript as narratives reflecting personal life experiences. In order to handle this issue properly, I consulted with my university's IRB staff member regarding the unique nature of the study and the active involvement of the CI users in the paper's development, thus meriting co-authorship. The IRB staff member considered the study to fall under the category of oral history of a unique sample, which does not fit under the regular stipulations for human subjects research. An important consideration is that these individuals are unique and do not represent a typical cohort of CI users, and this limits generalizaiton of their data. Also, they are sharing life experiences with full knowledge of their identity being

revealed and they are eager to do so in the spririt of self advocacy and increased public awareness.

The IRB staff member asked me to submit our plan of study through a Human Subjects Research Determination Form to formalize this proces. The IRB determined because the data represented oral history of a highly unique group of individuals, and cannot be generalized to a larger population, this project was exempt from human subjects requirements. We received a formal letter to that effect. This opened the door for sharing the identity of the CI users as co-authors. In addition, each CI user provided me with written documentation that they wish to have their identity revealed with regard to their personal characteristics (e.g., hearing history, musical background). Those documents have been submitted along with our manuscript.

However, in an effort to ensure that we have taken every ethical precaution, every CI user, prior to participating in data collection, also received an invitation to participate in a qualitative research study for adult CI users that had already been approved by the IRB of the first author's university. Thus, we have used three mechanisms to ensure that the involvement of all persons is appropriate and meets ethical guidelines.

I realize that as editors, you serve as guardians of the ethical practices for this special issue. I hope that this explanation will be helpful as you consider our submission.
